# Sleep Microstructure and Memory Function

**DOI:** 10.3389/fneur.2013.00159

**Published:** 2013-10-11

**Authors:** Luigi Ferini-Strambi, Andrea Galbiati, Sara Marelli

**Affiliations:** ^1^Sleep Disorders Center, Università Vita-Salute San Raffaele, Milan, Italy

**Keywords:** sleep microstructure, cyclic alternating pattern, memory, sleep spindles, cognitive functions

## Non-REM Sleep, REM Sleep, and Memory

Several studies investigated the effect of sleep on memory. In humans, arguably the first experimental description of a beneficial role of sleep for memory stabilization was provided in 1924 (1), indicating a protective benefit of sleep in preventing the normal decay-curve of forgetting that develops across time spent awake. Specific stages of sleep appear to be critical for memory consolidation. Memory is commonly divided into a declarative and a non-declarative memory system (2). Declarative memory is defined by memories accessible to conscious recollection, i.e., memories for events in a spatio-temporal context (episodic memory) and fact-based information (semantic memory). Procedural memory for skills is the type of non-declarative memory most thoroughly studied with regard to the effects of sleep. Sleep is characterized by the cyclic occurrence of REM and NREM sleep comprising SWS (sleep stages 3 and 4) and lighter sleep stages 1 and 2. Two hypotheses were proposed regarding sleep stages and memory consolidation (3). The dual process theory assumes that the specific sleep stages support consolidation of different types of memories. SWS supports declarative memory consolidation whereas REM sleep does so for procedural memories (4). The sequential hypothesis, on the other hand, proposes that sleep benefits memory optimally through the cyclic succession of both SWS and REM sleep. The original version of this hypothesis assumed that SWS functions to weaken non-adaptive memory traces whereas REM sleep re-stores the remaining traces (5). The dual process hypothesis received support mainly based on the early late sleep comparison, i.e., an approach comparing effects of retention intervals covering the first (SWS-rich) or the second (REM sleep-rich) half of nocturnal sleep. SWS-rich early sleep consistently found to support consolidation of hippocampus-dependent declarative memories, whereas REM sleep benefited non-declarative types of memory like priming, and memories for visuo-motor skills (6, 7). However, this dichotomy does not fit all results. Several non-declarative tasks, like visual texture discrimination, are also supported by SWS whereas REM sleep in some instances seems to benefit aspects of declarative memory (8).

More recently, Genzel and colleagues (9) tried to clarify the link between specific sleep stages and different types of memory consolidation by suppressing sleep stages. They deprived subjects once each of REM sleep and SWS, and once let them sleep undisturbed through the night. After each night, the authors tested declarative and procedural memory consolidation. Although REM sleep and SWS awakenings led to a significant reduction of the respective sleep stages, memory consolidation remained unaffected. According to the authors, there are two possible explanations for REM deprivation and SWS deprivation not influencing sleep-dependent consolidation of motor tasks: (1) the diminished amount of REM sleep in the REM deprivation condition was still sufficient for sleep-dependent memory consolidation or (2) the memory consolidation is dependent on stage 2 sleep. Another explanation of the results is that sleep-associated processes contributing to memory consolidation requires analyses of polysomnographic phenomena, like sleep spindles and slow-wave activity (SWA). This aspect is very important also for the implications for the cognitive decline by increasing age (10, 11). Other factors that change with age, such as hormones and neurotransmitters, or hypoxia related to sleep-disordered breathing (12, 13), may also play an important role.

## Sleep Spindles and Memory

Sleep spindles – a hallmark of stage 2 – are characterized in humans by waxing and waning 11- to 15-Hz oscillations lasting 0.5–3 s. They are generated by the thalamus, which acts as a pacemaker (14) and result from reciprocal rhythmic interactions between reticular and thalamo-cortical cells. Several reports described an association between NREM sleep spindle events and memory, including hippocampal-dependent learning. These short synchronous bursts of activity coincide with hippocampal sharp waves and ripples (15, 16), possibly reflecting reactivation of learned memory representations (17). Moreover, spindle density was associated with next-day memory recall: Meier-Koll and colleagues (18) reported a similar increase in spindles following learning of a hippocampally dependent maze task, and Clemens and colleagues (19) identified a correlation between spindle density and overnight verbal memory retention. The spindle-related memory processing is supported by very recent data showing that the same cortical areas active during learning are also (re)activated during post-encoding sleep spindle events (20). Sleep spindles can be separated into two subtypes based on frequency (21): “slow” (11–13 Hz) and “fast” (13–15 Hz). Concerning the memory consolidation, recent neuroimaging data demonstrated that the occurrence of fast sleep spindles coincide with moments of increased functional connectivity between the hippocampus and areas of neocortex (22), supporting a postulated component of the hippocampal-neocortical model of sleep-dependent memory consolidation. Indeed, several studies described select associations between episodic memory and fast- but not slow-spindle activity (23, 24). It is known that healthy aging is characterized by a reduced numbers of sleep spindles (25), and several studies reported reduced sleep spindles density in patients with Parkinson’s disease (26, 27).

## Cyclic Alternating Pattern

Increases in SWA was observed after learning a procedural rotation adaptation task and declarative word-pair learning (28). Increases in SWA after rotation adaptation learning were restricted to the motor cortical areas required for task acquisition. Inducing sleep slow oscillations (<1 Hz) by transcranial application of slowly oscillating potentials intensifies SWS and enhances consolidation of declarative memory (29): following learning of a word-pair list, transcranial direct current stimulation was applied over the prefrontal cortex of participants during early night SWS, inducting slow oscillation-like field potentials (in this case, at 0.75 Hz). Consequently, a greater benefit on overnight retention was observed for the set of information learned prior to sleep. Direct current stimulation not only increased the amount of slow oscillation activity, as well as sleep spindle frequency activity, during the simulation period (and for some time after), but also enhanced next-day word-pair retention. These findings were interpreted in the context of slow oscillations potentially triggering spindle-regulated plasticity in cortex. Slow EEG oscillations are the main components of the cyclic alternating pattern (CAP), that is expression of sleep microstructure. CAP is a periodic EEG activity during NREM sleep, characterized by sequences of abrupt frequency and amplitude variations (Phases A) followed by a return to background activity (Phases B). A CAP sequence is composed of a succession of CAP cycles. A CAP cycle is composed of a phase A and the following phase B. Phases A were subdivided into A1, A2, and A3 subtypes, based on the proportion of SWA and faster EEG (30). In subtype A1, EEG synchrony is the predominant activity and specimens include delta bursts, K-complex sequences, vertex sharp transients. In subtype A2, the EEG activity is a mixture of slow and fast rhythms with 20–50% of phase A occupied by EEG desynchrony. In subtype A3, the EEG activity is predominantly rapid low-voltage rhythms with >50% of phase A occupied by EEG desynchrony. Besides CAP, the other major EEG activity in the frequency range below 1 Hz is the so-called slow oscillation (31). This 0.5–0.9 Hz EEG rhythm, which characterizes states of reduced tonic arousal, was outlined during anesthesia and NREM sleep in animals and humans. The slow oscillation is generated in cortical neurons, and consists of phases of depolarization, characterized by intensive neural firing, followed by long-lasting hyperpolarization. The coalescence of slow rhythms is visible during NREM sleep, and slow waves converge into collectives resulting in the phase A1 subtypes of CAP. CAP A1 subtypes are almost exclusively composed by slow waves, map over the frontal regions of the scalp and correspond to periods in which the functional organization of the EEG network shows enhanced small-world network characteristics, a condition favorable for cognitive processing. These features make CAP A1 subtypes reliable candidates for a significant role in sleep-related cognitive processes. The first study supporting the idea that CAP slow components (A1) may be beneficial for cognitive processing was performed by Ferini-Strambi and colleagues (32), that evaluated a young individual with superior memory performance and found an increased % of CAP A1.

Since Huber and colleagues (33) found that a motor learning task performed during the day was followed by an increase in EEG SWA during the night and that SWA correlated with an improvement in subsequent performance, Ferri and colleagues (34) examined their original recordings and found that the night after the task, the CAP A1 subtypes were significantly increased in number and this increase correlated with improvements in task performance. Furthermore, in subjects with Asperger syndrome, significant correlations between verbal and non-verbal performance and CAP were found, with a general positive correlation with the A1 subtypes and a negative correlation with A2 and A3 subtypes (35). Moreover, dyslexic children have been reported to show a significant positive correlation between A1 subtypes and verbal and full-scale IQ, while CAP rate in NREM sleep stage 3 is positively correlated with verbal IQ (36). Thus, there is accumulating evidence implicating CAP as an important intermediate in cognitive processing. In another more recent publication, Aricò and colleagues (37) reported that CAP rate and A1 subtypes influence cognitive function in healthy subjects. The study illustrates the significant positive correlation between A1 subtypes and neurocognitive tests that characterize frontal lobe cognitive functions (e.g., verbal fluency, working memory, verbal learning). Furthermore, cognitive performance appears negatively correlated with A2 subtypes, whereas A3 subtypes are negatively correlated with planning and motor sequencing. These results provide additional support for the hypothesis that the A1 subtypes are associated with higher cognitive functioning, whereas the A2 and A3 subtypes are associated with impaired neurocognitive functioning. These results were confirmed by another more recent experimental study (38) that explored the association between CAP and neurocognitive performance in a group of normal subjects before and after two nights of experimentally induced sleep fragmentation. After sleep fragmentation, even if the percentage of SWS was dramatically reduced, there was a twofold increase in total CAP rate across all NREM sleep stages, and all phase A indexes significantly increased. Also this study supports that CAP A1 subtypes are associated with higher cognitive functioning, whereas CAP A3 subtypes are associated with lower cognitive functioning in young healthy subjects. Unfortunately, there are no systematic data on the relationship between CAP and cognitive function in healthy elderly, as well as in neurodegenerative disorders. The percentage of A1 subtypes shows clear changes by the age (39), with a decrease from teenagers (71%) to young adults (61%) and middle age (62%), and a marked decline after age 60 (46%). In contrast, subtypes A2 and A3 undergo a linear increase from infants, pre-school children to the old age, similar to the arousal evolution across the life span. A recent study (40) evaluated CAP in patients with idiopathic REM sleep behavior disorder (iRBD), a parasomnia that may be an early manifestation of a neurodegenerative disease. CAP sequences and CAP rate were significantly higher in iRBD patients compared to the controls. The increase in CAP sequences was observed in phase A2 and A3 subtypes while phase A1 subtype was significantly lower in RBD patients. This finding is interesting since a neurocognitive impairment was described in iRBD patients (41). In conclusion, a consistent literature demonstrated the relationship between sleep and memory, and in particular the need for sleep after learning in the consolidation of hippocampal-dependent memory. The importance of the first half of the night for declarative memory consolidation originates from sleep spindles and slow EEG oscillations. These distinct polysomnographic phenomena characterizing sleep microstructure show a decrease as aging progresses.
